# Hydrothermal evolution, optical and electrochemical properties of hierarchical porous hematite nanoarchitectures

**DOI:** 10.1186/1556-276X-8-2

**Published:** 2013-01-02

**Authors:** Wancheng Zhu, Xili Cui, Xiaofei Liu, Liyun Zhang, Jia-Qi Huang, Xianglan Piao, Qiang Zhang

**Affiliations:** 1 Department of Chemical Engineering, Qufu Normal University, Shandong, 273165, China; 2 Department of Chemical Engineering, Tsinghua University, Beijing, 100084, China

**Keywords:** Hematite, Hierarchical nanoarchitectures, Hydrothermal, Mesoporous, Lithium-ion batteries

## Abstract

Hollow or porous hematite (α-Fe_2_O_3_) nanoarchitectures have emerged as promising crystals in the advanced materials research. In this contribution, hierarchical mesoporous α-Fe_2_O_3_ nanoarchitectures with a pod-like shape were synthesized via a room-temperature coprecipitation of FeCl_3_ and NaOH solutions, followed by a mild hydrothermal treatment (120°C to 210°C, 12.0 h). A formation mechanism based on the hydrothermal evolution was proposed. β-FeOOH fibrils were assembled by the reaction-limited aggregation first, subsequent and *in situ* conversion led to compact pod-like α-Fe_2_O_3_ nanoarchitectures, and finally high-temperature, long-time hydrothermal treatment caused loose pod-like α-Fe_2_O_3_ nanoarchitectures via the Ostwald ripening. The as-synthesized α-Fe_2_O_3_ nanoarchitectures exhibit good absorbance within visible regions and also exhibit an improved performance for Li-ion storage with good rate performance, which can be attributed to the porous nature of Fe_2_O_3_ nanoarchitectures. This provides a facile, environmentally benign, and low-cost synthesis strategy for α-Fe_2_O_3_ crystal growth, indicating the as-prepared α-Fe_2_O_3_ nanoarchitectures as potential advanced functional materials for energy storage, gas sensors, photoelectrochemical water splitting, and water treatment.

## Background

Three-dimensional hierarchical architectures, or nanoarchitectures, assembled by one-dimensional (1D) nanostructures have attracted extraordinary attention and intensive interests owing to their unique structures and fantastic properties different from those of the monomorph structures [1-5]. Particularly, hierarchical architectures with mesoporous structures have triggered more and more research enthusiasm in recent years for their high surface-to-volume ratio and permeability. Synthesis of mesoporous materials has become a remarkable level in modern materials chemistry [6]. Mesoporous materials are generally synthesized via a soft- or hard-template-aided process, which usually, however, suffers from the removal of templates and resultant structural collapse, although hydrothermal synthesis or treatment has been extensively investigated at various stages with the attempt to improve the hydrothermal stability of the as-synthesized mesoporous products. Consequently, great effort has been made to directly grow mesoporous inorganic materials in the absence of any templates in recent years [7,8]. Most recently, the hydrothermal method has emerged as a thriving technique for the facile fabrication of the nanoarchitectures [9-12], such as AlOOH cantaloupe [13], Co(OH)_2_ and Co_3_O_4_ nanocolumns [14], ZnSe nanoflowers [15], Ni(OH)_2_ and NiO microspheres [16], and even mesoporous SrCO_3_ microspheres [8].

As the most stable iron oxide, hematite (α-Fe_2_O_3_) has drawn much concern owing to its widespread applications as catalysts, pigments, gas sensors [17], photoelectrodes [17,18], starting materials for the synthesis of magnetic iron oxide nanoparticles (NPs) [19], electrode materials for lithium-ion battery (LIB) [20-26], etc. α-Fe_2_O_3_ is considered a promising active lithium intercalation host due to its high theoretical capacity (1,007 mAh·g^−1^), low cost, and environmental friendliness. In contrast to graphite electrodes, the lithium storage within iron oxides is mainly achieved through the reversible conversion reaction between lithium ions and metal nanocrystals dispersed in a Li_2_O matrix [24]. Such a process usually causes drastic volume changes (>200%) and severe destruction of the electrode upon electrochemical cycling, especially at a high rate [24]. Particle morphology has been recognized as a key factor influencing the electrochemical performance for lithium storage; thus, hematite nanostructures with different morphologies have been synthesized so as to enhance the electrochemical performance [22]. The mesoporous α-Fe_2_O_3_ nanoarchitectures may afford several advantages for LIB application, such as the extended contact area between the active material and the electrolyte as well as the short lithium diffusion length resulting from the thin shell and the hollow space in the central part that buffers the volume expansion during cycling [22,27,28].

Up to now, a family of hierarchical α-Fe_2_O_3_ architectures (microring [7], melon-like [25], columnar [29], and nanotube [30] arrays; nanoplatelets [31]; peanut- [32], cantaloupe- [33], or urchin-like [34] nanoarchitectures, etc.) have been available. Most recently, novel hollow architectures (hollow fibers [35], hollow particles [36], hollow microspheres and spindles [37,38], etc.) and porous nanoarchitectures (nanoporous microscale particles [39], mesoporous particles [40,41], nanocrystal clusters [42], porous nanoflowers [43], etc.) have emerged as the new highlights in crystal growth. However, hollow or porous hematite nanoarchitectures were generally fabricated via a forced hydrolysis (100°C, 7 to 14 days) reaction [40], surfactant-assisted solvothermal process [38,42], and hydrothermal- [37] or solvothermal-based [43] or direct [42] calcination (400°C to 800°C) methods. The reported methodologies exhibited drawbacks such as ultralong time or high energy consumption and potentially environmental malignant. It was still a challenge to directly acquire porous/mesoporous hematite nanoarchitectures via a facile, environmentally benign, and low-cost route.

In our previous work, we developed a hydrothermal synthesis of the porous hematite with a pod-like morphology or short-aspect-ratio ellipsoidal shape (denoted as ‘pod-like’ thereafter) in the presence of H_3_BO_3_[44]. However, the process still needed to be optimized, the formation mechanism and the effect of H_3_BO_3_ were not clear, and properties and potential applications also needed to be further investigated. In this contribution, we report our newly detailed investigation on the optimization of the process and formation mechanism of the mesoporous nanoarchitectures based on the hydrothermal evolution. In addition, the effect of H_3_BO_3_ was discussed, the optical and electrochemical properties of the as-synthesized hematite mesoporous nanoarchitectures as well as nanoparticles were investigated in detail, and the application of the as-synthesized mesoporous hematite nanoarchitectures as anode materials for lithium-ion batteries was also evaluated.

## Methods

### Hydrothermal synthesis of the hierarchical hematite nanoarchitectures

All reagents, such as FeCl_3_·6H_2_O, NaOH, and H_3_BO_3_, were of analytical grade and used as received without further purification. Monodisperse α-Fe_2_O_3_ particles were synthesized via a coprecipitation of FeCl_3_ and NaOH solutions at room temperature, followed by a facile hydrothermal treatment of the slurry in the presence of H_3_BO_3_ as the additive. In a typical procedure, 1.281 g of H_3_BO_3_ was poured into 10.1 mL of deionized (DI) water, then 9.3 mL of FeCl_3_ (1.5 mol·L^−1^) solution was added, and finally 7.0 mL of NaOH (4 mol·L^−1^) solution was dropped into the above mixed solution under vigorous magnetic stirring at room temperature, with the molar ratio of FeCl_3_/H_3_BO_3_/NaOH as 2:3:4. After 5 min of stirring, 26.4 mL of the resultant brown slurry was transferred into a Teflon-lined stainless steel autoclave with a capacity of 44 mL. The autoclave was sealed and heated to 90°C to 210°C (heating rate 2°C·min^−1^) and kept under an isothermal condition for 1.0 to 24.0 h, and then cooled down to room temperature naturally. The product was filtered, washed with DI water for three times, and finally dried at 80°C for 24.0 h for further characterization. To evaluate the effects of the molar ratio of the reactants, the molar ratio of FeCl_3_/H_3_BO_3_/NaOH was altered within the range of 2:(0–3):(2–6), with other conditions unchanged.

### Evaluation of the hematite nanoarchitectures as the anode materials for lithium batteries

The electrochemical evaluation of the Fe_2_O_3_ NPs and nanoarchitectures as anode materials for lithium-ion batteries were carried out using CR2025 coin-type cells with lithium foil as the counter electrode, microporous polyethylene (Celgard 2400, Charlotte, NC, USA) as the separator, and 1.0 mol·L^−1^ LiPF_6_ dissolved in a mixture of ethylene carbonate, dimethyl carbonate, ethylene methyl carbonate (1:1:1, by weight) as the electrolyte. All the assembly processes were conducted in an argon-filled glove box. For preparing working electrodes, a mixed slurry of hematite, carbon black, and polyvinylidene fluoride with a mass ratio of 80:10:10 in *N*-methyl-2-pyrrolidone solvent was pasted on pure Cu foil with a blade and was dried at 100°C for 12 h under vacuum conditions, followed by pressing at 20 kg·cm^−2^. The galvanostatic discharge/charge measurements were performed at different current densities in the voltage range of 0.01 to 3.0 V on a Neware battery testing system (Shenzhen, China). The specific capacity was calculated based on the mass of hematite. Cyclic voltammogram measurements were performed on a Solartron Analytical 1470E workstation (Farnborough, UK) at a sweep rate of 0.1 mV·s^−1^.

### Characterization

The crystal structures of the samples were identified using an X-ray powder diffractometer (XRD; D8-Advance, Bruker, Karlsruhe, Germany) with a Cu K_α_ radiation (*λ* = 1.5406 Å) and a fixed power source (40.0 kV, 40.0 mA). The morphology and microstructure of the samples were examined using a field-emission scanning electron microscope (SEM; JSM 7401 F, JEOL, Akishima-shi, Japan) operated at an accelerating voltage of 3.0 kV. The size distribution of the as-synthesized hierarchical architectures was estimated by directly measuring *ca*. 100 particles from the typical SEM images. The N_2_ adsorption-desorption isotherms were measured at 77 K using a chemisorption-physisorption analyzer (Autosorb-1-C, Quantachrome, Boynton Beach, FL, USA) after the samples had been outgassed at 300°C for 60 min. The specific surface area was calculated from the adsorption branches within the relative pressure range of 0.10 to 0.31 using the multipoint Brunauer-Emmett-Teller (BET) method, and the pore size distribution was evaluated from the N_2_ desorption isotherm using the Barrett-Joyner-Halenda method. The optical properties were examined using a UV–vis spectrophotometer (Cary 300, Varian, Palo Alto, CA, USA), with absolute alcohol as the dispersive medium.

## Results and discussion

### Hematite structures obtained at different molar ratios of the reactants

**Figure 1:**
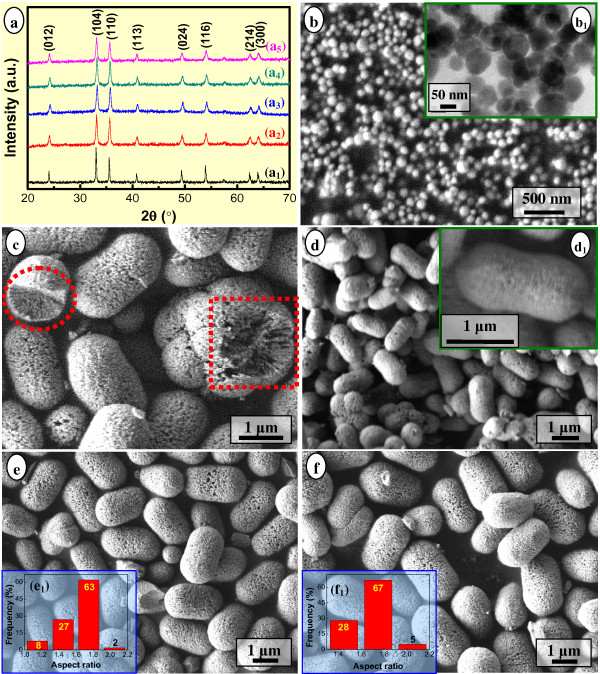
**XRD patterns (a) and SEM (b, c-f) and TEM (b_1_) images of the hydrothermal products.** The products were obtained at 150°C for 12.0 h with different molar ratios of FeCl_3_/H_3_BO_3_/NaOH = 2:0:6 (a_1_, b, b_1_), 2:0:4 (a_2_, c), 2:0:2 (a_3_, d, d_1_), 2:0.3:4 (a_4_, e, e_1_), 2:1.5:4 (a_5_, f, f_1_). Inset: aspect ratio distributions of the corresponding samples (e_1_, f_1_).

Figure 1 shows the influences of the molar ratio of FeCl_3_/H_3_BO_3_/NaOH on the compositions and morphologies of the hydrothermal products obtained at 150°C for 12.0 h. When changing the molar ratio of FeCl_3_/H_3_BO_3_/NaOH within the range of 2:(0–3):(2–6), all products were composed of pure-phase hematite (α-Fe_2_O_3_, JCPDS No. 33–0664), with a detectable slight difference of the crystallinity (Figure 1a). With the molar ratio of FeCl_3_/H_3_BO_3_/NaOH changed from 2:0:6 to 2:0:4 and to 2:0:2, the crystallinity of hematite decreased slightly (Figure 1a_1_,a_2_,a_3_). In contrast, the morphologies of the obtained products varied significantly with the change of the molar ratio of reactants. Quasi-spherical hematite NPs with a diameter of 30 to 150 nm were obtained when the molar ratio of FeCl_3_/H_3_BO_3_/NaOH was 2:0:6 (Figure 1b,b_1_), similar to the so-called α-Fe_2_O_3_ nanopolyhedra synthesized in the ammonia-water system at 180°C for 8.0 h [23]. With the molar ratio decreased to 2:0:4 and 2:0:2, hierarchical pod-like (with elliptical ends and relatively uniform diameter along the long axial direction, Figure 1c) and peanut-type nanoarchitectures (with relatively sharp elliptical ends and saddle-shaped middle part, Figure 1d,d_1_) were acquired, respectively. The pod-like architectures contained 1D or linear chain-like assemblies of smaller nanoparticles or rod-like subcrystals within the body (as shown in red dotted elliptical and rectangular regions in Figure 1c), with distinct cavities on the surfaces (Figure 1c). The peanut-type nanoarchitectures (Figure 1d,d_1_) also comprised small nanoparticles within the body whereas with not so distinct cavities on the surfaces owing to the relatively compact assembly. Similar 1D assemblies, such as rod-like subcrystals and linear chains of interconnected primary particles, have also been found to exist as the subunits of peanut-type [45] and double-cupola [46] hematite, respectively. Obviously, the molar ratio of 2:0:6 (FeCl_3_/H_3_BO_3_/NaOH) led to nearly monodisperse hematite NPs, whereas the molar ratio of 2:0:4 and 2:0:2 resulted in porous hierarchical architectures with different morphologies. According to Sugimoto’s research [45,47,48], size control is generally performed by controlling the number of nuclei during the nucleation stage, and nucleation occurs during the addition of NaOH solution into FeCl_3_ solution. In the present case, the molar ratio of FeCl_3_/H_3_BO_3_/NaOH as 2:0:6 is the stoichiometric ratio for the formation of colloidal Fe(OH)_3_ at room temperature, which led to the greatest degree of supersaturation of Fe(OH)_3_ and further resulted in the largest number of nuclei and ultimately brought the quasi-spherical α-Fe_2_O_3_ NPs.

However, when H_3_BO_3_ was introduced into the reaction system, e.g., the molar ratio of FeCl_3_/H_3_BO_3_/NaOH was designed as 2:0.3:4 (Figure 1a_4_,e,e_1_) and 2:1.5:4 (Figure 1a_5_,f,f_1_), relatively uniform porous pod-like hematite nanoarchitectures were obtained. For the ratio of 2:0.3:4, 90% of the nanoarchitectures have an aspect ratio (ratio of longitudinal length to latitude diameter) within 1.4 to 1.8 (Figure 1e_1_). For the hematite obtained from a molar ratio of FeCl_3_/H_3_BO_3_/NaOH as 2:1.5:4, 95% of the nanoarchitectures have an aspect ratio within 1.4 to 1.8 (Figure 1f_1_). Therefore, the introduction of H_3_BO_3_ not only preserved the shape of hematite particles, but also improved the morphology uniformity of the nanoarchitectures. This situation was different from that of the formation of peanut-type hematite, which evolved from pseudocubic particles via an ellipsoidal shape with the increasing concentration of the additive such as sulfate or phosphate [49]. On the other hand, compared with those organic surfactant-assisted solvothermal or other solution-based calcination methods, the introduced H_3_BO_3_ in the present case could be easily removed via DI water washing and then reused, indicating the environmentally benign characteristic.

### Effects of hydrothermal temperature on the hematite product formation

**Figure 2:**
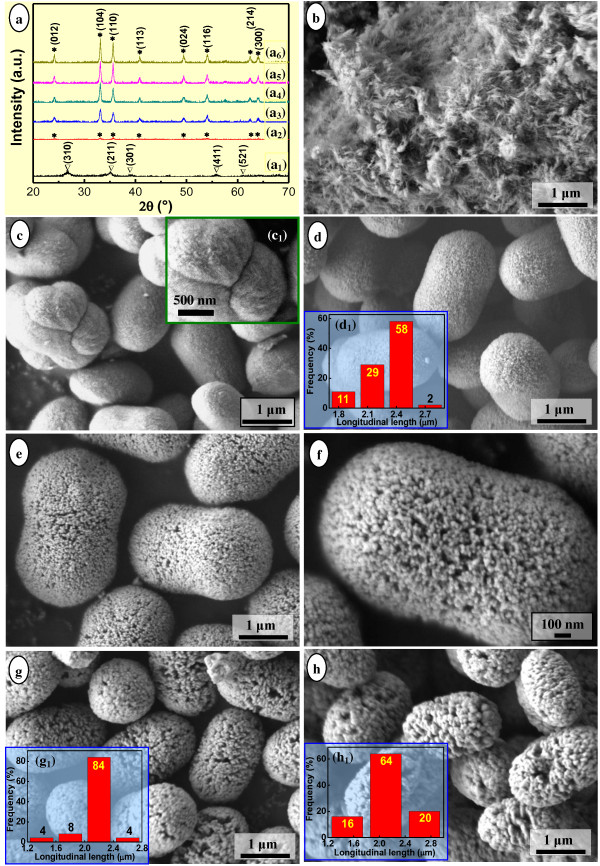
**XRD patterns (a) and SEM images (b-h) of the hydrothermal products.** The products were synthesized at different temperatures for 12.0 h, with the molar ratio of FeCl_3_/H_3_BO_3_/NaOH = 2:3:4. Temperature (°C) = 90 (a_1_, b), 105 (a_2_, c), 120 (a_3_, d), 150 (a_4_, e, f), 180 (a_5_, g), 210 (a_6_, h). Inset: high-resolution SEM image (c_1_) as well as the longitudinal length distributions (d_1_, g_1_, h_1_) of the corresponding samples. The asterisk represents hematite (α-Fe_2_O_3_, JCPDS No. 33–0664); nabla represents akaganeite (β-FeOOH, JCPDS No. 34–1266).

The compositions and morphologies of the hydrothermal products obtained at various temperatures for 12.0 h were tracked so as to further understand the corresponding evolution, as shown in Figure 2. Obviously, the hydrothermal temperature had significant influences on the compositions as well as the morphologies of the products. The sample hydrothermally treated at 90°C was composed of relatively poor-crystallinity and low-aspect-ratio akaganeite (β-FeOOH, JCPDS No. 34–1266, Figure 2a_1_) nanorods or nanofloccules (Figure 2b). When hydrothermally treated at 105°C, the product gradually changed into poor-crystallinity α-Fe_2_O_3_ (Figure 2a_2_, JCPDS No. 33–0664) of pod-like and pumpkin-like nanoarchitectures (Figure 2c). Moreover, the local details showed that the nanoarchitecture consisted of short 1D nanostructured subunits and tiny NPs (Figure 2c_1_). When treated at 120°C, α-Fe_2_O_3_ nanoarchitectures with greatly improved crystallinity (Figure 2a_3_) and uniform compact pod-like morphology (Figure 2d) were formed, 87% of which had a longitudinal length of 2.2 to 2.5 μm (Figure 2d_1_). Notably, the compact nanoarchitecture contained numerous tiny NPs onto the surfaces. With the temperature increasing up to 150°C and keeping it constant for 12.0 h, the products comprised uniform porous pod-like α-Fe_2_O_3_ with higher crystallinity (Figure 2a_4_) and multitudinal cavities on the surfaces (Figure 2e,f), 84% of which had a longitudinal length of 2.6 to 3.2 μm [44]. The morphology of the present pod-like α-Fe_2_O_3_ nanoarchitectures was somewhat similar to that of the melon-like microparticles by the controlled H_2_C_2_O_4_ etching process [25]. With the temperature further going up to 180°C, porous pod-like α-Fe_2_O_3_ nanoarchitectures with further improved crystallinity (Figure 2a_5_) and more and larger cavities on the surfaces were obtained (Figure 2g), 84% of which had a longitudinal length of 2 to 2.4 μm (Figure 2g_1_). When hydrothermally treated at 210°C for 12.0 h, the product evolved into high-crystallinity whereas entirely loose porous α-Fe_2_O_3_ nanoarchitectures (Figure 2a_6_,h), 84% of which had a longitudinal length of 2.1 to 2.7 μm (Figure 2h_1_).

It was worth noting that when treated at a temperature from 90°C to 210°C for 12.0 h, the overall crystallinity of the products became higher (Figure 2a_2_,a_3_,a_4_,a_5_,a_6_), and the NPs and cavities within the α-Fe_2_O_3_ nanoarchitectures grew larger. The product evolved from compact pod-like nanoarchitectures (Figure 2c,d) to loose (Figure 2e,f) and to looser (Figure 2g,h) pod-like nanoarchitectures. As a matter of fact, with the temperature going up from 120°C to 150°C, to 180°C, and to 210°C, the crystallite size along the [104] direction, i.e., *D*_104_, calculated by the Debye-Scherrer equation also increased from 23.3 to 27.3, to 28.0, and to 31.3 nm, respectively. This was in accordance with the direct observation on the gradual increase in the NP size within the nanoarchitectures (Figure 2d,e,f,g,h), thus accounted for the gradual sharper tendency for the XRD patterns of the corresponding hydrothermal products (Figure 2a_3_,a_4_,a_5_,a_6_) obtained from 120°C to 210°C. Analogous to those obtained previously (Figure 1c,e,f), the nanoarchitectures obtained at 150°C to 210°C for 12.0 h were speculated to be constituted of 1D assemblies (Figure 2e,f) or NPs (Figure 2g,h).

### Determination of the mesoporous structure of the pod-like α-Fe_2_O_3_ nanoarchitectures

**Figure 3:**
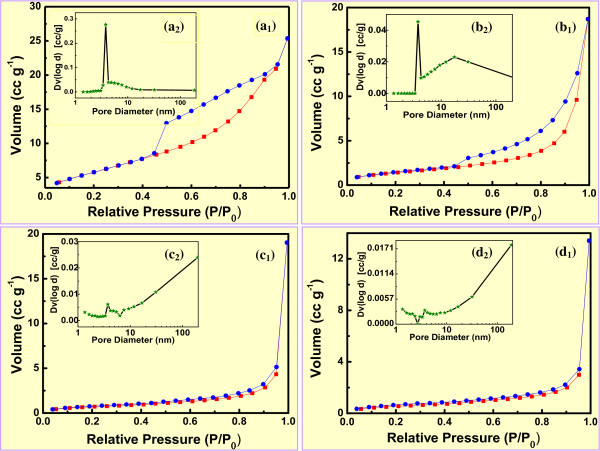
**Nitrogen adsorption-desorption isotherms (a_1_-d_1_) and corresponding pore diameter distributions (a_2_-d_2_) of the mesoporous α-Fe_2_O_3_.** The nanoarchitectures were synthesized at different temperatures for 12.0 h, with the molar ratio of FeCl_3_/H_3_BO_3_/NaOH = 2:3:4. Temperature (°C) = 120 (a_1_, a_2_); 150 (b_1_, b_2_); 180 (c_1_, c_2_); 210 (d_1_, d_2_). The blue line with blue circles represents the desorption curve; the red line with square rectangles represents the adsorption curve.

Figure 3 shows the N_2_ adsorption-desorption isotherms and corresponding pore size distributions of the hydrothermally synthesized α-Fe_2_O_3_ nanoarchitectures with typical morphologies. Influences of the temperature on the porous α-Fe_2_O_3_ nanoarchitectures are summarized in Table 1. As listed, the selected nanoarchitectures 1, 2, 3, and 4 corresponded with those obtained at 120°C (Figure 2d), 150°C (Figure 2e,f), 180°C (Figure 2g), and 210°C (Figure 2h) for 12.0 h, respectively. All N_2_ adsorption-desorption isotherms of the nanoarchitectures exhibited type IV with an H3-type hysteresis loop. The compact pod-like nanoarchitecture 1 (Figure 2d, *D*_104_ = 23.3 nm) had a relatively large adsorbance of N_2_ (Figure 3a_1_) with a broad hysteresis loop at a relative pressure *P*/*P*_0_ of 0.45 to 0.95 and a very narrow pore diameter distribution concentrating on 3.8 nm (Figure 3a_2_). In contrast, the relative loose pod-like nanoarchitecture 2 (Figure 2e,f, *D*_104_ = 27.3 nm) showed a relatively small adsorbance of N_2_ (Figure 3b_1_) with a typical H3-type hysteresis loop at a relative pressure *P*/*P*_0_ of 0.45 to 1.0 and a bimodal pore diameter distribution concentrating on 3.8 and 17.5 nm (Figure 3b_2_). The characteristic N_2_ adsorption-desorption isotherms (Figure 3a_1_,b_1_) and pore size distributions (Figure 3a_2_,b_2_) revealed that both nanoarchitectures 1 and 2 are of mesoporous structures.

**Table 1:** Mesoporous structures of the α-Fe_2_O_3_ synthesized at different temperatures for 12.0 h (FeCl_3_/H_3_BO_3_/NaOH = 2:3:4)

**α-Fe_2_O_3_ nanoarchitecture**	**Temperature**	**Multipoint BET**	**Total pore volume**	**Average pore diameter**
	**(°C)**	**(m^2^ g^−1^)**	**(cm^3^ g^−1^)**	**(nm)**
1	120	21.3	3.9 × 10^−2^	7.3
2	150	5.2	2.9 × 10^−2^	22.1
3	180	2.6	2.9 × 10^−2^	44.7
4	210	2.0	2.1 × 10^−2^	40.3

Comparatively, the looser pod-like nanoarchitecture 3 (Figure 2g, *D*_104_ = 28.0 nm) demonstrated a similar adsorbance of N_2_ (Figure 3c_1_) whereas with a narrow hysteresis loop at a relative pressure *P*/*P*_0_ of 0.40 to 0.95 and a quasi-bimodal pore diameter distribution (Figure 3c_2_). Very similarly, the loosest pod-like nanoarchitecture 4 (Figure 2h, *D*_104_ = 31.3 nm) exhibited a relatively low adsorbance of N_2_ (Figure 3d_1_) with also a narrow hysteresis loop at a relative pressure *P*/*P*_0_ of 0.25 to 0.95 as well as a quasi-bimodal pore diameter distribution (Figure 3d_2_). It was worth noting that the broad hysteresis loop (Figure 3a_1_) and relative narrow one (Figure 3b_1_) were due to the strong and weak capillarity phenomena existing within the compact (Figure 2d) and relatively loose nanoarchitectures (Figure 2e), respectively. Moreover, the characteristic H3-type hysteresis loop (Figure 3b_1_) indicated the existence of dominant slit pores and channels with a relatively uniform shape and size within the relatively loose pod-like nanoarchitectures (Figure 2e,f). This was in accordance with the SEM observation (Figure 1c) and literature results [45,46]. The thin hysteresis loops (Figure 3c_1_,d_1_) were due to the slight capillarity phenomenon existing within the very loose nanoarchitectures (Figure 2g,h).

As shown in Table 1, with the temperature increasing from 120°C to 150°C, to 180°C, and to 210°C, the corresponding multipoint BET specific surface area of the nanoarchitecture decreased from 21.3 to 5.2, to 2.6, and to 2.0 m^2^·g^−1^, respectively. Meanwhile, the total pore volume changed from 3.9 × 10^−2^ to 2.9 × 10^−2^, to 2.9 × 10^−2^, and to 2.1 × 10^−2^ cm^3^·g^−1^, with a roughly decreasing tendency; the average pore diameter changed from 7.3 to 22.1, to 44.7, and to 40.3 nm, with a roughly increasing tendency. Thus, according to the general recognition of the porous materials [50], nanoarchitectures 3 and 4 were determined as the mesoporous structures, whereas the pore diameters were near the macropores category. As a matter of fact, with the temperature increasing from 120°C to 210°C, the evolution of the BET specific surface area, total pore volume, and average pore diameter of the various-morphology pod-like α-Fe_2_O_3_ nanoarchitectures agreed with the variation of the *D*_104_ calculated by the Debye-Scherrer equation, also in accordance with the SEM observation (Figure 2d,e,f,g,h).

### Evolution of the hydrothermal products during hydrothermal process

**Figure 4:**
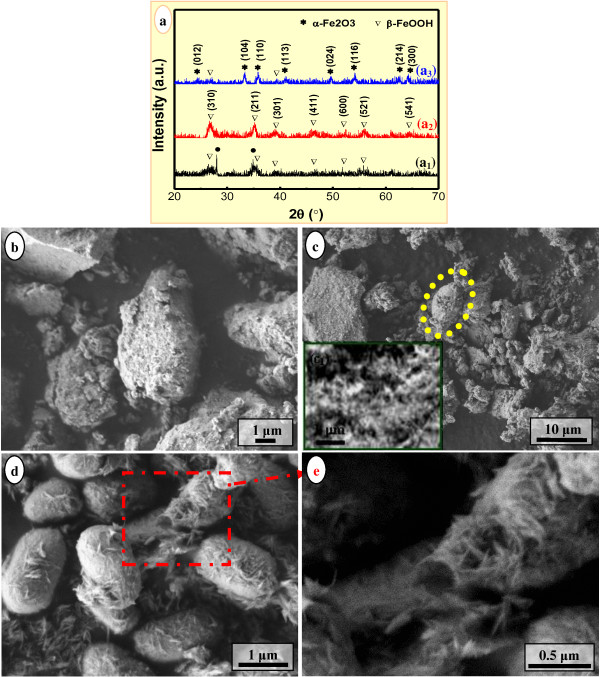
**Composition (a) and morphology (b-e) evolution of the hydrothermal products.** The products were obtained at 105°C for different times, with the molar ratio of FeCl_3_/H_3_BO_3_/NaOH = 2:3:4. Time (h) = 1.0 (a_1_, b); 3.0 (a_2_, c); 6.0 (a_3_, d, e). The asterisk represents α-Fe_2_O_3_ (JCPDS No. 33–0664); nabla represents β-FeOOH (JCPDS No. 34–1266); the bullet represents maghemite (γ-Fe_2_O_3_, JCPDS No. 25–1402). Inset: high-resolution SEM image of the corresponding sample (c_1_).

Since the compact pod-like nanoarchitecture obtained at 105°C for 12.0 h (Figure 2c) bridged 1D β-FeOOH nanostructures and pod-like α-Fe_2_O_3_ nanoarchitectures, the composition and morphology of the products hydrothermally treated at 105°C for various times were monitored, as shown in Figure 4. All hydrothermal products obtained at 105°C for 1.0 to 12.0 h exhibited relatively poor crystallinity (Figure 4a_1_,a_2_,a_3_). When treated for 1.0 h, the product was composed of β-FeOOH (JCPDS No. 34–1266) and detectable trace amount of maghemite (γ-Fe_2_O_3_, JCPDS No. 25–1402) in a nearly amorphous state (Figure 4a_1_,b). With the time extending to 3.0 h, the product was only β-FeOOH with improved crystallinity, and γ-Fe_2_O_3_ no longer existed (Figure 4a_2_,c). Notably, β-FeOOH at that period exhibited very tiny primary 1D morphology (i.e., fibrils, Figure 4c_1_), and a rudimental pod-like aggregate was also observed (denoted as yellow dotted elliptical region in Figure 4c). When treated for 6.0 h, the hydrothermal products containing trace amount of β-FeOOH and majority of newly formed α-Fe_2_O_3_ (Figure 4a_3_ were acquired, exhibiting pod-like or ellipsoidal-shaped aggregates entangled with 1D nanostructures (Figure 4d). The enlarged image (Figure 4e) corresponding to the red dot-dashed rectangular region in Figure 4d clearly showed that the selected developing pod-like aggregate was assembled by 1D β-FeOOH nanowhiskers. In other words, the pod-like aggregate did not simply coexist or was not simply coated with, but constructed by 1D β-FeOOH nanostructures. With the time prolonged to 12.0 h, as mentioned previously, the pure phase of α-Fe_2_O_3_ nanoarchitectures consisted of very tiny NPs with compact pod-like and pumpkin-like morphologies acquired (Figure 2a_2_,c). The crystallite size *D*_104_ calculated by the Debye-Scherrer equation was 20.5 nm, smaller than that of the compact pod-like α-Fe_2_O_3_ nanoarchitectures obtained at 120°C for 12.0 h (Figure 2d) due to a relatively lower temperature hydrothermal treatment.

### Formation mechanism of mesoporous pod-like α-Fe_2_O_3_ nanoarchitectures

From the phase conversion and morphology evolution of the hydrothermal products, formation of the monodisperse pod-like α-Fe_2_O_3_ phase could be further clarified, which experienced a two-step phase transformation from Fe(OH)_3_ to β-FeOOH and from β-FeOOH to α-Fe_2_O_3_[51,52]. The room-temperature coprecipitation of FeCl_3_ and NaOH solutions and hydrolysis of excessive Fe^3+^ ions can be expressed as

(1) $$\begin{array}{l} {\mathrm{Fe}}^{3+}\left(\mathrm{aq}\right)\\ \;+{3\mathrm{OH}}^{-}\left(\mathrm{aq}\right)\to \mathrm{Fe}{\left(\mathrm{OH}\right)}_{3}\left(\mathrm{amorphous}\;\mathrm{gel}\right) \end{array}$$

(2) $$\begin{array}{l} {\mathrm{Fe}}^{3+}\left(\mathrm{aq}\right)\\ \;+{3H}_{2}O\to \mathrm{Fe}{\left(\mathrm{OH}\right)}_{3}\left(\mathrm{amorphous}\;\mathrm{gel}\right)+{3\;H}^{+}\left(\mathrm{aq}\right) \end{array}$$

Hydrothermal conversion of amorphous Fe(OH)_3_ gel can be expressed as

(3) $$\mathrm{Fe}{\left(\mathrm{OH}\right)}_{3}\left(\mathrm{gel}\right)\to \beta -\mathrm{FeOOH}\left(\mathrm{fibrils}\right)+H_{2}O$$

(4) $$\begin{array}{l} 2\beta -\mathrm{FeOOH}\left(\mathrm{fibrils}\right)\to \alpha -{\mathrm{Fe}}_{2}O_{3}\left(\mathrm{mesoporous}\right)\\ \;+H_{2}O \end{array}$$

As known, iron oxyhydroxides (FeOOH) can be crystallized as goethite (α-FeOOH), lepidocrocite (γ-FeOOH), and akaganeite (β-FeOOH), and an environment rich of Cl^−^ was favorable for the formation of β-FeOOH phase [53]. In the present case, a molar ratio of the reactants as FeCl_3_/H_3_BO_3_/NaOH = 2:(0–3):4 led to a surrounding rich of Cl^−^ and thus promoted the formation of β-FeOOH. Tiny β-FeOOH fibrils with poor crystallinity formed at the early stage of the hydrothermal treatment (e.g., 90°C, 12.0 h, Figure 2a_1_; 105°C, 1.0 to 3.0 h, Figure 4a_1_,a_2_) tended to agglomerate with each other owing to the high surface energy, leading to quasi-amorphous agglomerate bulks of irregular shape (Figures 2b and 4b,c). Undoubtedly, the conversion from β-FeOOH to α-Fe_2_O_3_ was crucial to the formation of mesoporous pod-like hematite nanoarchitectures. Sugimoto et al. reported a preparation of monodisperse peanut-type α-Fe_2_O_3_ particles from condensed ferric hydroxide gel in the presence of sulfate [49] and found that ellipsoidal hematite turned into a peanut-like shape with the increase in the concentration of sulfate [51]. In the present case, although quasi-spherical α-Fe_2_O_3_ NPs were obtained in due case (Figure 1b), the mesoporous hematite nanoarchitectures (Figures 1c,d,e,f and 2d,e,f,g,h) were not directly assembled by those NPs, taking into consideration the remarkable differences of the morphology especially size between the NPs and subunits of nanoarchitectures. It was worth noting that the hydrothermally formed hematite particles exhibited a peanut-like shape at the molar ratio of FeCl_3_/H_3_BO_3_/NaOH as 2:0:2 (Figure 1d) and a pod-like shape at the molar ratio of FeCl_3_/H_3_BO_3_/NaOH as 2:(0–3):4 (Figures 1c,e,f and 2d,e,f,g,h). Moreover, with the content of H_3_BO_3_ increasing, the pod-like α-Fe_2_O_3_ nanoarchitectures tended to be uniform in size distribution. Consequently, the morphology evolution of the hydrothermally synthesized α-Fe_2_O_3_ nanoarchitectures in the presence of boric acid, from a peanut-type to a pod-like shape, was obviously different from that of the peanut-type α-Fe_2_O_3_ particles that originated from condensed ferric hydroxide gel in the presence of sulfate [49].

**Figure 5:**
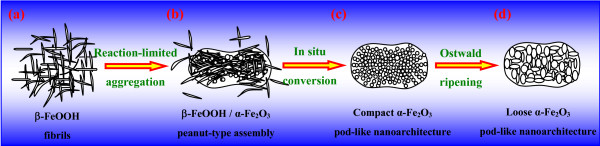
Formation mechanism of the hierarchical mesoporous pod-like hematite nanoarchitectures.

Thus, based on the present experimental results (Figures 1, 2, 3, and 4), the overall formation mechanism of mesoporous pod-like hematite nanoarchitectures in the presence of boric acid was illustrated in Figure 5. Firstly, the amorphous Fe(OH)_3_ gel derived from room-temperature coprecipitation was hydrothermally treated under an environment rich of Cl^−^, leading to poor-crystallinity β-FeOOH fibrils (Figure 5a) [53]. Secondly, with the hydrothermal temperature going up and time going on, β-FeOOH fibrils were organized into a peanut-type assembly, and at the same time, β-FeOOH fibrils began to dissolve, resulting in α-Fe_2_O_3_ NPs. As a consequence, peanut-like β-FeOOH/α-Fe_2_O_3_ assemblies were obtained (Figure 5b). This process was very analogous to the ‘rod-to-dumbbell-to-sphere’ transformation phenomenon, which had been found in the formation of some other hierarchical architectures, such as carbonates (CaCO_3_, BaCO_3_, SrCO_3_, MnCO_3_, CdCO_3_) [8,54,55], fluoroapatite (Ca_5_(PO_4_)_3_OH) [56], etc. Like the dumbbell transition structure, the present peanut-type assembly was also believed to be formed due to the reaction-limited aggregation. Thirdly, with the hydrothermal treatment further going on, remanent β-FeOOH fibrils were further dissolved and the peanut-like β-FeOOH/α-Fe_2_O_3_ assemblies were converted into relatively compact pod-like α-Fe_2_O_3_ nanoarchitectures, consisting of 1D or linear chain-like assemblies of rod-like subcrystals or tiny NPs within the body (Figure 5c). No proof convinced that the peanut-type β-FeOOH/α-Fe_2_O_3_ assemblies were thoroughly dissolved and reorganized into the pod-like nanoarchitectures with almost unchanged external shape and size. In other words, peanut-like β-FeOOH/α-Fe_2_O_3_ assemblies were *in situ* transformed into α-Fe_2_O_3_ NPs within the peanut-like aggregates owing to the hydrothermal treatment. However, the *in situ* converted tiny α-Fe_2_O_3_ NPs bore high surface energy. This promoted the aggregation, instead of the segregation, of those tiny NPs so as to reduce the overall surface energy, leading to relatively compact pod-like α-Fe_2_O_3_ nanoarchitectures due to a slight expansion of the entire volume. Finally, with the hydrothermal treatment going on, the compact pod-like α-Fe_2_O_3_ nanoarchitectures became looser and looser owing to the coarsening [57,58] of the constitutional NPs controlled by the traditional Ostwald ripening, i.e., dissolution-reprecipitation mechanism (Figure 5d) [58]. The constitutional α-Fe_2_O_3_ subcrystals grew into larger NPs, with 1D assembly behavior disappeared largely.

It is notable, however, that the boric acid played a significant role in the formation of the present mesoporous pod-like α-Fe_2_O_3_ nanoarchitectures with uniform morphology and size, confirmed by the above experimental results (Figures 1 and 2). Also, as confirmed to improve the uniformity, the amount of boric acid or molar ratio of FeCl_3_/H_3_BO_3_/NaOH should be tuned within a certain composition range. As known, as a weak acid, H_3_BO_3_ could form sodium borate (i.e., borax) after the introduction of NaOH, giving rise to the buffer solution. This could tune the release of hydroxyl ions and further control the mild formation of amorphous Fe(OH)_3_ gel, leading to subsequent β-FeOOH fibrils with relatively uniform size. This was believed to contribute to the further formation of the peanut-like β-FeOOH/α-Fe_2_O_3_ assemblies and ultimate occurrence of the pod-like α-Fe_2_O_3_ nanoarchitectures.

### Optical absorbance analysis

**Figure 6:**
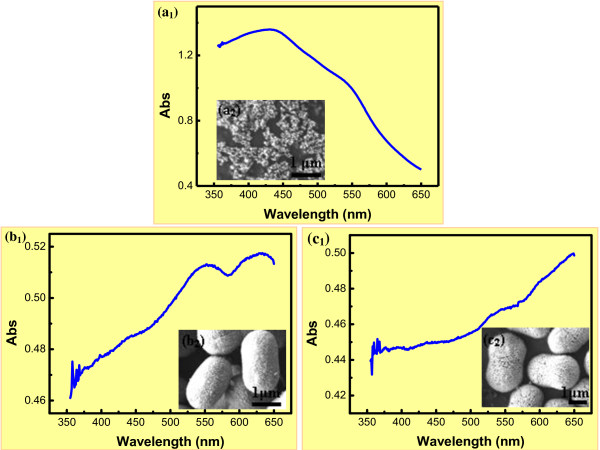
**Optical absorbance spectra (a_1_-c_1_) of the α-Fe_2_O_3_ with different morphologies (a_2_-c_2_).** Time (h) = 12.0; Temperature (°C) = 120 (a_1_, a_2_, b_1_, b_2_), 150 (c_1_, c_2_); FeCl_3_/H_3_BO_3_/NaOH = 2:3:6 (a_1_, a_2_), 2:3:4 (b_1_, b_2_, c_1_, c_2_).

Hematite NPs have been widely used as ultraviolet absorbents for their broad absorption in the ultraviolet region from the electron transmission of Fe-O. Figure 6 shows the optical absorbance spectra of the α-Fe_2_O_3_ particles with the photon wavelength in the range of 350 to 650 nm. For sample a_1_, it revealed two absorption edges around 380 to 450 and 540 to 560 nm, which were consistent with the reported hematite NPs [59-61]. When the α-Fe_2_O_3_ clustered into samples b_1_ and c_1_, the size of α-Fe_2_O_3_ agglomerates was around 500 to 800 nm. The absorbance spectra showed two absorption peaks around 520 to 570 and 600 to 640 nm. The change in the degree of transition depended on the shape and size of the particles. When the hematite particles aggregated to pod-like nanoarchitectures, the size became larger, and then the scattering of visible light was superimposed on the absorption of as-prepared architectures.

It was well illustrated that three types of electronic transitions occurred in the optical absorption spectra of Fe^3+^ substances: (a) the Fe^3+^ ligand field transition or the *dd* transitions, (b) the ligand to metal charge-transfer transitions, and (c) the pair excitations resulting from the simultaneous excitations of two neighboring Fe^3+^ cations that are magnetically coupled. According to [62,63], the absorption bands near 390 and 430 nm corresponded to the ^6^A_1_ → ^4^E(^4^G) and ^6^A_1_ → ^4^E, ^4^A_1_(^4^G) ligand field transitions of Fe^3+^[59,60]. The observed edge at around 520 to 570 and 600 to 640 nm could be assigned to the ^6^A_1_ → ^4^ T_2_(^4^G) ligand field transition of Fe^3+^. As revealed by Figure 6, the electronic transition for the charge transfer in the wavelength region 380 to 450 nm dominated the optical absorption features of the NPs, while the ligand field transitions in the range of 520 to 640 nm dominated the optical absorption features of the architectures. This indicated that the absorption could be modulated by controlling the size and shape of the hematite, which was quite important for the enhancement of the photoelectrocatalytic activity.

### Mesoporous pod-like α-Fe_2_O_3_ nanoarchitectures as anode materials for lithium-ion batteries

**Figure 7:**
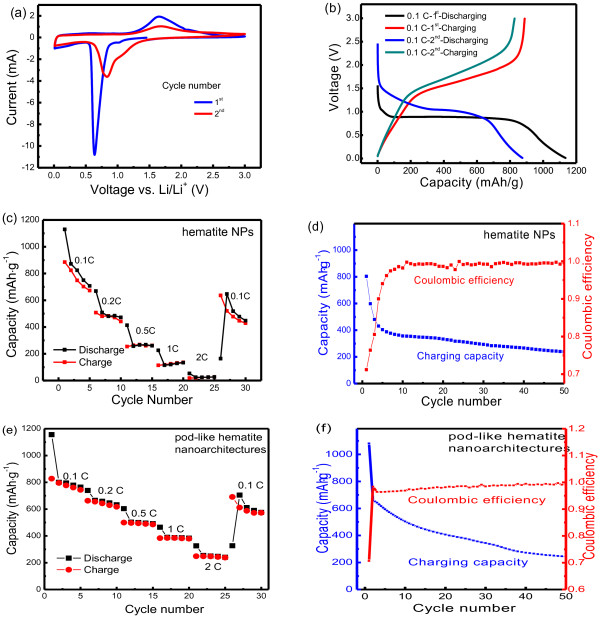
**Representative cyclic voltammograms and charge–discharge performances of the hematite electrode.** (**a**) Representative cyclic voltammograms of the hematite nanoparticles (presented in Figure 1b) at a scan rate of 0.1 mV s^−1^; (**b**) the charge–discharge performances at various current rates (1 C = 1,006 mA g^−1^, corresponding to the full discharge in 1 h, a rate of *n* C corresponds to the full discharge in 1/*n* h) of the hematite nanoparticles; (**c**) the rate performance and (**d**) the cycling performance at a current of 1 C of an electrode fabricated with the hematite nanoparticles presented in Figure 1b; (**e**) the rate performance and (**f**) the cycling performance at a current of 1 C of an electrode fabricated with hierarchical mesoporous pod-like hematite nanoarchitectures presented in Figure 2e.

The electrochemical behavior of the hematite electrodes was evaluated by cyclic voltammetry and galvanostatic charge/discharge cycling. As shown in Figure 7a, a spiky peak was observed at 0.65 V with a small peak appearing at 1.0 V during the cathodic polarization of the hematite NPs (presented in Figure 1b) in the first cycle. This indicated the following lithiation steps [43,64,65]:

(5) $$\alpha -{\mathrm{Fe}}_{2}O_{3}+{2\mathrm{Li}}^{+}+{2e}^{-}\to {\mathrm{Li}}_{2}{\mathrm{Fe}}_{2}O_{3}\left(\mathrm{cubic}\right)$$

(6) $${\mathrm{Li}}_{2}{\mathrm{Fe}}_{2}O_{3}\left(\mathrm{cubic}\right)+{4\mathrm{Li}}^{+}+{4e}^{-}\to {2\mathrm{Fe}}^{0}+{3\mathrm{Li}}_{2}O$$

With lithium ions inserted into the crystal structure of the as-prepared α-Fe_2_O_3_, the hexagonal α-Fe_2_O_3_ was transformed to cubic Li_2_Fe_2_O_3_. The peak at 0.65 V corresponded to the complete reduction of iron from Fe^2+^ to Fe^0^ and the decomposition of electrolyte. A broad anodic peak was recorded in the range of 1.4 to 2.2 V, corresponding to the oxidation of Fe^0^ to Fe^2+^ and further to Fe^3+^[66,67]. The curve of the subsequent cycle was significantly different from that of the first cycle as only one cathodic peak appeared at about 0.8 V with decreased peak intensity, while the anodic process only showed one broad peak with a little decrease in peak intensity. The irreversible phase transformation during the process of lithium insertion and extraction in the initial cycle was the reason for the difference between the first and second cathodic curves [24]. After the first discharge process, α-Fe_2_O_3_ was completely reduced to iron NPs and was dispersed in a Li_2_O matrix. The decrease of the redox peak intensity implied that the capacity was decreased during cycling.

The charge–discharge curves of the α-Fe_2_O_3_ NP (shown in Figure 1b) electrode during the first and second cycles are shown in Figure 7b. In the first discharge curve, there was a weak potential slope located at 1.2 to 1.0 V and an obvious potential plateau at 0.9 to 0.8 V. The capacity obtained above 0.8 V was 780 mAh·g^−1^ (4.6 mol of Li per mole of α-Fe_2_O_3_). After discharging to 0.01 V, the total specific capacity of the as-prepared α-Fe_2_O_3_ reached 887 mAh·g^−1^, corresponding to 5.3 mol of Li per mole of α-Fe_2_O_3_. During the second cycle, the discharge curve only showed a slope at 1.0 to 0.8 V, and the capacity was reduced to 824 mAh·g^−1^. Usually, the slope behavior during the discharge process of metal oxide anode materials was considered to be related with the irreversible formation of a nanocomposite of crystalline grains of metals and amorphous Li_2_O matrix.

The comparison of the rate as well as cycling performances between Fe_2_O_3_ NPs and nanoarchitectures were also conducted, which were obtained by a 12.0-h hydrothermal treatment at 150°C with a molar ratio of FeCl_3_/H_3_BO_3_/NaOH as 2:0:4 (Figure 1b) and 2:3:4 (Figure 2e), respectively. The discharge and charge capacities in the first cycle at a current of 0.1 C were 1,129 and 887 mAh·g^−1^ for Fe_2_O_3_ NPs (Figure 7c) and 1,155 and 827 mAh·g^−1^ for Fe_2_O_3_ nanoarchitectures. For the second cycle, the discharge and charge capacities were 871 and 824 mAh·g^−1^ for Fe_2_O_3_ NPs and 799 and 795 mAh·g^−1^ for Fe_2_O_3_ nanoarchitectures. The Li-ion storage capacitance of the current Fe_2_O_3_ NPs/nanoarchitectures reported in this work is higher than that of hematite nanorod (*ca*. 400 mAh·g^−1^ at 0.1 C) [68], nanoflakes [69], hierarchial mesoporous hematite (*ca*. 700 mAh·g^−1^ at 0.1 C) [65], hollow nanospindles (457 mAh·g^−1^ at 0.2 mA cm^−2^) [37], hollow microspheres (621 mAh·g^−1^ at 0.2 mA cm^−2^) [37], and dendrites (670 mAh·g^−1^ at 1 mA cm^−2^) [70]. When the current increased, both the discharge and charge capacities decreased, especially for Fe_2_O_3_ NPs (Figure 7c,e). The discharge and charge capacities of Fe_2_O_3_ nanoarchitectures were larger than those of Fe_2_O_3_ NPs. For instance, when the current rate increased to 2.0 C, the charge and discharge capacities of Fe_2_O_3_ nanoarchitectures were 253 and 247 mAh·g^−1^, while those of Fe_2_O_3_ NPs were only 24 and 21 mAh·g^−1^. This indicated that the Fe_2_O_3_ nanoarchitectures presented much improved rate performance for the reason that the porous nature of Fe_2_O_3_ nanoarchitectures allow a fast Li-ion diffusion by offering better electrolyte accessibility and also accommodate the volume change of NPs during Li insertion/extraction.

However, similar to many Fe_2_O_3_ nanostructures reported in literatures, the α-Fe_2_O_3_ nanoarchitectures exhibited a rapid capacity fading within the potential range of 0.01 to 3.0 V, suggesting that the crystalline structure of the electrode materials was destroyed by the insertion/extraction of lithium ions and the electrode decomposed the electrolyte. The Fe_2_O_3_ nanoarchitectures presented superior charge/discharge stability to the Fe_2_O_3_ NPs, e.g., the charging capacities of Fe_2_O_3_ nanoarchitectures (Figure 7f) and NPs (Figure 7d) of the tenth cycle were 503 and 356 mAh·g^−1^, respectively. This indicated that the mesoporous structure of Fe_2_O_3_ nanoarchitectures provided more space for Fe_2_O_3_ volume change and avoided severe pulverization. Such an improvement could also be confirmed by the cycling performance of mesoporous hematite [67], which maintained a good stability attributed from the small Fe_2_O_3_ size (*ca*. 10 nm) and abundant pores. The introduction of conductive carbon into the hematite electrode is an effective way to improve the cycle performance [68]. It is highly expected that the hierarchical Fe_2_O_3_ nanoarchitectures with ultrafine Fe_2_O_3_ building blocks and interconnected pores afford shorter Li-ion diffusion way, fast diffusion rate, and large-volume changes during the charge/discharge process, which can serve as potential anode materials for Li-ion storage.

## Conclusions

Uniform monodisperse hierarchical α-Fe_2_O_3_ nanoarchitectures with a pod-like shape have been synthesized via a facile, environmentally benign, and low-cost hydrothermal method (120°C to 210°C, 12.0 h), by using FeCl_3_·6H_2_O and NaOH as raw materials in the presence of H_3_BO_3_ (molar ratio, FeCl_3_/H_3_BO_3_/NaOH = 2:3:4). The mesoporous α-Fe_2_O_3_ nanoarchitectures had a specific surface area of 21.3 to 5.2 m^2^·g^−1^ and an average pore diameter of 7.3 to 22.1 nm. The mesoporous α-Fe_2_O_3_ nanoarchitectures were formed as follows: the reaction-limited aggregation of β-FeOOH fibrils led to β-FeOOH/α-Fe_2_O_3_ peanut-type assembly, which was subsequently and *in situ* converted into compact pod-like α-Fe_2_O_3_ nanoarchitectures and further into loose pod-like α-Fe_2_O_3_ nanoarchitectures through a high-temperature, long-time hydrothermal treatment via the Ostwald ripening. Benefiting from their unique structural characteristics, the as-synthesized hierarchical mesoporous pod-like α-Fe_2_O_3_ nanoarchitectures exhibited good absorbance and a high specific discharge capacity. Compared with the traditional solid-state monomorph hematite NPs and other complicated porous hematite nanoarchitectures, the as-synthesized hierarchical mesoporous pod-like α-Fe_2_O_3_ nanoarchitectures derived from the facile, environmentally benign, and low-cost hydrothermal route can provide an alternative candidate for novel applications in booming fields, such as gas sensors, lithium-ion batteries, photocatalysis, water treatment, and photoelectrochemical water splitting.

## Competing interests

The authors declare that they have no competing interests.

## Authors’ contributions

WCZ provided guidance to XLC, XFL, and LYZ as he was the supervisor. WCZ and QZ wrote the paper. JQH conducted the research study on the Li-ion storage performance test. XLP conducted the surface area measurement. All authors read and approved the final manuscript.
